# The association between smoking behaviour, social cognition and social functioning in patients with a non-affective psychotic disorder: A prospective follow-up study

**DOI:** 10.1016/j.scog.2021.100206

**Published:** 2021-06-30

**Authors:** Tobias E.G. Dekker, Heleen Susanne van der Heijden, Frederike Schirmbeck, Therese van Amelsvoort, Agna A. Bartels-Velthuis, Claudia J.P. Simons, Lieuwe de Haan, Jentien M. Vermeulen

**Affiliations:** a Early Psychosis Department, Amsterdam UMC (location AMC), Department of Psychiatry, Amsterdam, the Netherlands; b Arkin Institute for Mental Health, Amsterdam, the Netherlands; c Maastricht University Medical Center, Department of Psychiatry and Neuropsychology, School for Mental Health and Neuroscience, Maastricht, the Netherlands; d University of Groningen, University Medical Center Groningen, University Center for Psychiatry, Rob Giel Research Center, Groningen, the Netherlands; e GGzE Institute for Mental Health Care, Eindhoven, the Netherlands

**Keywords:** Tobacco, Emotion processing, Theory of mind, Schizophrenia

## Abstract

**Introduction:**

In patients with psychotic disorders, both tobacco smoking and deficits in social cognition and social functioning are highly prevalent. However, little is known about their relationship in psychosis. The authors sought to evaluate the multi-cross-sectional and longitudinal associations between tobacco smoking, social cognition and social functioning in a large prospective study.

**Methods:**

This study was performed within the Genetic Risk and Outcome of Psychosis (GROUP) Study, a cohort study conducted in patients with non-affective psychosis (N = 1074), their unaffected siblings (N = 1047) and healthy controls (N = 549). At baseline, three years and six years of follow-up, data on tobacco smoking (using the Composite International Diagnostic Review), social cognition (emotion processing and theory of mind) and social functioning were collected. To assess associations between tobacco smoking and social cognition or social functioning, multivariate linear mixed-effects models and multiple linear regression models were used. Bonferroni correction for multiple testing was applied.

**Results:**

A significant positive association was found between smoking and emotion processing (as part of social cognition) in the patient group (estimate = 1.96, SE = 0.6, p = 0.003). However, smoking was significantly negatively associated with participating in pro-social activities compared with non-smoking (estimate = −2.55, SE = 0.9, p = 0.004). Change in smoking behaviour was not associated with social cognition or social functioning in the longitudinal analyses.

**Conclusion:**

Findings indicate that smoking patients with a non-affective psychotic disorder slightly outperformed their non-smoking peers on a task on social cognition, but participated less in pro-social activities. Commencement or cessation of smoking was not related to social cognition or functioning.

## 1 Introduction

Adults with a psychotic disorder have a higher lifetime prevalence of tobacco smoking than the general population (de Leon and Diaz, 2005). Furthermore, the prevalence of smoking cessation among patients with psychosis has been found to be lower compared to healthy controls and patients with other psychiatric disorders (Zeng et al., 2020). Frequency of smoking in the general population has decreased in the past few decades but this has not been true for patients with a psychotic disorder (Faith Dickerson et al., 2013). A prominent feature of individuals with psychosis is reduced cognitive performance including social cognition (Savla et al., 2013). The latter is introduced as one of seven impaired cognitive domains in the Measurement and Treatment Research to Improve Cognition in Schizophrenia (MATRICS) (Nuechterlein et al., 2004). Social cognition and neurocognitive domains are relatively independent constructs (Pinkham et al., 2003), though have been found to be correlated. For example, processing socially relevant information relies on neurocognition (e.g., attention or memory) (Fett et al., 2011). Moreover, social cognition mediates the relationship between neurocognition and social functioning, making it an important driver of functional outcomes and overall recovery in patients with schizophrenia (Javed and Charles, 2018). Social cognition is defined as the mental processes by which humans interpret and respond to others' behaviour (Niendam et al., 2009) and is closely related to social functioning (Fett et al., 2011). For example, facial affect recognition (which is often impaired in psychosis (Kohler et al., 2010)) is an important aspect of social functioning (Hofer et al., 2009).

Smoking, social cognition and social functioning are entangled in a complex triangle. Acute nicotine administration has been suggested to have enhancing effects on social cognition and functioning (Martin and Sayette, 2018). However, this relationship is far from straightforward. For example, Niemegeers et al. (2014) found that in the general population, acute nicotine administration improved social functioning in those with poor baseline social functioning, but had negative effects on those with higher baseline functioning. In patients with psychosis, Quisenaerts et al. (2013) found that acute nicotine improved social decision-making in non-smoking patients, but not in smoking patients. Adolescent tobacco use in the general population was associated with poor social communication and poor social reciprocity later in life (Fluharty et al., 2018). To date, very few studies have assessed the impact of long-term nicotine smoking on social cognition and functioning in psychosis. One large cross-sectional study evaluating 335 patients with first-episode psychosis showed no significant differences in social cognition between smoking and non-smoking patients (Sánchez-Gutiérrez et al., 2018), nor did another cross-sectional study (Reed et al., 2016) that assessed social cognition in 76 smoking and non-smoking patients with a psychotic disorder. With respect to social functioning, no differences were found between smoking and non-smoking patients with therapy-resistant schizophrenia (Iasevoli et al., 2013).

To the best of our knowledge, longitudinal studies evaluating the relationship between smoking, social cognition and social functioning are lacking. This is unfortunate, considering the well-known detrimental effects of chronic smoking and the important role of social cognition and social functioning in patients with a psychotic disorder. Therefore, this prospective study investigated associations between tobacco smoking, social cognition and social functioning in a sample of patients with non-affective psychosis, unaffected siblings and healthy controls with a follow-up of six years. The primary aim was to evaluate whether smoking status or the number of cigarettes smoked per day were associated with social cognition or social functioning. Second, we aimed to explore whether change in smoking status or change in number of cigarettes smoked per day were associated with change in social cognition or social functioning. Due to the absence of longitudinal studies evaluating long-term smoking behaviour, social cognition and functioning in patients with psychosis, no specific hypotheses were formulated.

## 2 Methods

### 2.1 Study sample and design

This study was performed within the Genetic Risk and Outcome of Psychosis (GROUP) study, which is a multi-site longitudinal cohort study involving patients with a non-affective psychotic disorder, their unaffected siblings and healthy control subjects. For patients, inclusion criteria were an age range of 16 to 50 years old and a diagnosis of non-affective psychosis according to the DSM-IV (Diagnostic and Statistical Manual of Mental Disorders: DSM-IV-TR. Washington: American Psychiatric Publisher). Siblings were included in the patient group and controls were excluded if afflicted with a non-affective psychotic disorder. Further study details can be found elsewhere (Korver et al., 2012). Assessments were performed at baseline, three years and six years of follow-up (supplement 1). Written informed consent was acquired from all participants before the first assessment. The study was approved by the Medical Ethics Committee of the Academic Medical Centre of Utrecht.

### 2.2 Measurements

#### 2.2.1 Smoking

To determine the degree of tobacco smoking, the Composite International Diagnostic Interview (Cottler et al., 1989) was used at all three assessments. It allows evaluation of the quality and severity of substance dependence and its course over time. The cross-cultural acceptability and reliability of the questions were found to be high in a field trial (Cottler et al., 1989). Participants were defined as smokers if they had smoked tobacco on a daily basis for at least one month in the past 12 months. Data on smoking cigars or on chewing and snuffing tobacco were excluded.

#### 2.2.2 Social cognition

Three instruments were used to assess two domains of social cognition (Pinkham et al., 2014): emotion processing (i.e. the ability to infer emotional information from facial expressions (Couture et al., 2006)) and theory of mind (ToM) (i.e. the ability to represent the mental states of others). For emotion processing, the computerized Degraded Facial Affect Recognition (DFAR) task was used (total score = 64) (van't Wout et al., 2004). The task shows faces of two male and two female actors expressing the following emotions: happy, angry, fearful and neutral. Participants were instructed to indicate which emotion applied to each face. The score on the DFAR represents the total percentage of correct answers. It was assessed at baseline and three years of follow-up.

For ToM, the Hinting task measured the ability to infer real intentions behind indirect speech utterances (total score = 20) (Corcoran et al., 1995; van Hooren et al., 2008). This task consists of 10 short passages presenting an interaction between two characters, of which one drops an obvious hint. Participants were asked what was meant by this hint. The Hinting task was assessed at baseline only. Notably, evidence shows that the Hinting task not only measures ToM, but verbal reasoning and immediate verbal learning and memory as well (Mallawaarachchi et al., 2019).

The Picture Sequencing Task (PST) was used as a second measure of ToM (total score = 24) (Langdon and Coltheart, 1999). Subjects were asked to sequence stories, by turning over the cards in front of them and placing them in the logical order. In false-belief stories (PST-FB), subjects were tested on their ability to predict that others can act on the basis of beliefs that misrepresent reality. Underperformance in patients with schizophrenia has previously been found for this outcome (Langdon et al., 2006). The PST was only assessed at six years of follow-up.

#### 2.2.3 Social functioning

The Social Functioning Scale (SFS) was used to measure the self-rated degree of social functioning. The SFS has shown to be reliable, valid and sensitive in a sample of patients with early psychosis and schizophrenia (Birchwood et al., 1990; Chan et al., 2019). In line with previous research (Schneider et al., 2017), four out of seven domains were selected: social engagement/withdrawal, interpersonal behaviour, recreational and pro-social activities. The SFS was assessed at three and at six years of follow-up.

### 2.3 Covariates

Based on existing literature (Depp et al., 2015; Hickling et al., 2018) and associations between smoking, cognition and functioning, the following covariates were selected a priori: age, gender, years of education as a proxy for socioeconomic status, cannabis use, antipsychotic medication use, severity of psychopathology and premorbid functioning. Cannabis use was evaluated with urine analysis at all assessments. In patients, current use of antipsychotic medication was registered. Moreover, severity of psychopathology was assessed using the Positive and Negative Syndrome Scale (PANSS) (Kay et al., 1987), of which the positive syndrome scale, the negative syndrome scale and the general psychopathology scale were used. The Community Assessment of Psychic Experiences (CAPE) was self-rated by siblings and controls to assess lifetime psychotic and depressive experiences (Mossaheb et al., 2012). The subscales for positive symptoms, negative symptoms and depressive symptoms were included. The Premorbid Adjustment Scale (PAS) was used to correct for premorbid functioning (Cannon-Spoor et al., 1982). The subscale of the first life period (up to 11 years old) was included.

### 2.4 Statistical analyses

Baseline differences in demographic and clinical characteristics and outcomes between smoking and non-smoking participants were tested with student *t*-tests, Mann-Whitney-*U* tests and Pearson chi-square tests.

Linear mixed-effects models were performed to evaluate the multi-cross-sectional association between smoking status and respectively the DFAR task and the included domains of the SFS. As fixed effects, smoking status and covariates were added. As random effects, intercepts for subjects and random slopes for time were entered. If a significant association was found between smoking status and the outcome variable, post hoc analyses were performed with number of cigarettes smoked per day.

Furthermore, multiple linear regression models were used to evaluate the cross-sectional association between smoking status and respectively the Hinting task and the PST. Post-hoc analyses were performed if a significant association was found between smoking status and outcome variables. If significant estimates were found in analyses for the DFAR task and the SFS domains (multi-cross-sectional), multiple linear regression models were run to assess the association between change in smoking behaviour and change in social cognition or functioning (see supplement 9).

Moreover, correlations between social cognition and social functioning were assessed using Spearman's correlation. No correlation coefficients were calculated for the Hinting task and SFS, since their assessment periods did not overlap. If a significant correlation was found, partial correlations were performed to assess a possible moderating effect of smoking behaviour.

Since seven different outcome variables were tested (DFAR, Hinting, PST and four scales of SFS), we used a Bonferroni correction to minimize the risk of type I errors. Thus, the two-tailed significance threshold was set at 0.007 (0.05/7). For the current study, release 7.00 of the GROUP database and SPSS version 26.0 were used for the analyses.

## 3 Results

### 3.1 Study sample demographics

At baseline, data on smoking status were available for 1.074 patients, 1.047 siblings and 549 healthy controls (see supplement 2). Differences in demographics are summarized in Table 1. Baseline characteristics of social cognition and functioning of smoking and non-smoking participants are listed in Table 2. See supplement 3 for further details on missing data on outcome variables.

**Table 1:** Baseline demographic characteristics of smoking and non-smoking participants.

	Patients (n = 1074)			Siblings (n = 1047)			Controls (n = 549)		
	Smoking 725 (67.5%)	Non- smoking 349 (32.5%)	p-value*	Smoking 401 (38.3%)	Non-smoking 646 (61.7%)	p-value*	Smoking 139 (25.3%)	Non-smoking 410 (74.7%)	p-value*
Age	26.7 (6.7)	28.2 (8.2)	0.014	27.5 (8.1)	28.1 (8.4)	0.175	28.9 (9.4)	30.1 (10.5)	0.401

Gender									
Male	602 (83.0%)	225 (64.5%)	<0.0001	198 (49.4%)	281 (43.5%)	0.063	70 (50.4%)	190 (46.3%)	0.412
Female	123 (17.0%)	124 (35.5%)		203 (50.6%)	365 (56.5%)		69 (49.6%)	220 (53.7%)	
Education in years	12.0 (3.7)	13.2 (3.9)	<0.0001	12.8 (3.9)	13.9 (4.0)	<0.0001	14.1 (3.1)	14.7 (3.4)	0.116

PANSS									
Positive subscale	1.9 (0.8)	1.7 (0.7)	<0.0001						
Negative subscale	2.0 (0.9)	1.9 (0.8)	0.257						
General subscale	1.8 (0.5)	1.7 (0.5)	<0.0001						

CAPE									
Positive symptoms				0.2 (0.2)	0.2 (0.2)	0.016	0.2 (0.2)	0.2 (0.2)	0.029
Negative symptoms				0.6 (0.4)	0.5 (0.4)	<0.0001	0.5 (0.3)	0.5 (0.3)	0.081
Depressive symptoms				0.7 (0.4)	0.6 (0.4)	0.002	0.6 (0.4)	0.6 (0.3)	0.076
PAS <12	1.4 (0.9)	1.4 (1.0)	0.576	1.0 (0.8)	1.0 (0.8)	0.218	0.9 (0.7)	1.1 (0.8)	0.086
Tested positive for cannabis	147 (23.0%)	6 (1.9%)	<0.0001	62 (17.5%)	11 (1.9%)	<0.0001	19 (14.3%)	8 (2.0%)	<0.0001
Antipsychotic drug use	616 (85.0%)	300 (86.0%)	0.667						

Data are n (%) or mean (SD). PANSS=Positive and Negative Syndrome Scale. CAPE = Community Assessment of Psychic Experience. Frequency subscales. PAS <12 = Premorbid Adjustment Scale. *Two-sided p values were computed by a *t*-test, a Mann-Whitney *U* test or a Pearson's χ^2^ test.

**Table 2:** Baseline characteristics of social cognition and functioning of smoking and non-smoking participants.

	Patients (n = 1074)		Siblings (n = 1047)		Controls (n = 549)	
	Smoking 725 (67.5%)	Non- smoking 349 (32.5%)	Smoking 401 (38.3%)	Non-smoking 646 (61.7%)	Smoking 139 (25.3%)	Non-smoking 410 (74.7%)
DFAR % total correct	69.1 (10.5)	67.3 (10.8)	73.1 (9.1)	72.1 (9.4)	74.2 (8.1)	72.9 (9.4)
Hinting total score	17.4 (2.9)	17.8 (2.5)	18.8 (1.8)	18.9 (1.6)	19.0 (1.4)	19.1 (1.2)
PST – false belief score^a^	19.1 (4.7)	18.7 (5.0)	20.3 (3.6)	20.2 (3.6)	20.3 (3.3)	20.0 (4.2)

SFS – scaled scores^a^						
Withdrawal	103.5 (12.5)	105.8 (11.2)	115.4 (11.5)	118.3 (11.3)	116.9 (12.0)	120.0 (10.5)
Interpersonal	123.8 (19.4)	125.1 (19.6)	137.4 (13.6)	139.3 (11.8)	139.4 (12.4)	141.2 (9.7)
Recreation	113.6 (15.9)	116.9 (14.7)	121.8 (14.2)	124.4 (13.5)	124.6 (13.7)	126.5 (12.1)
Pro-social	111.9 (13.8)	114.8 (13.8)	121.0 (11.4)	121.0 (11.6)	124.1 (10.9)	123.5 (9.8)

Data are in n (%) or mean (SD). DFAR = Degraded Facial Affect Recognition. PST = Picture Sequencing Task. SFS=Social Functioning Scale.

a Data shown for the PST was assessed at six-year follow-up, for the SFS at three-year follow-up.

### 3.2 Cross-sectional correlations between social cognition and social functioning

Cross-sectional correlations were performed between the DFAR and four domains of the SFS on three years of follow-up. For patients, siblings or controls, no significant correlations were found. Furthermore, correlations were assessed between the PST and four domains of the SFS on six years of follow-up (see supplement 4). A positive significant correlation between interpersonal behaviour and the PST was found for patients (r = 0.124, p = 0.003) and for healthy controls (r = 0.147, p = 0.007). Moreover, a negative correlation was found for recreational activities in siblings (r = −0.117, p = 0.003). Partial correlations were performed in order to assess a possible moderating effect of smoking behaviour (see supplement 5). This had very little or no influence on the strength of the correlation between the PST and the SFS domains in patients, siblings or healthy controls.

### 3.3 Multi-cross-sectional associations between smoking status and emotion processing

We evaluated multi-cross-sectional associations between smoking and emotion processing, assessed with the DFAR task, while correcting for covariates (Table 3). In patients, a significant positive association was found between smoking status and the score on the DFAR task (estimate = 1.96, SE = 0.6, p = 0.003). Post-hoc analysis revealed a significant association between number of cigarettes smoked per day and the DFAR task in patients (estimate = 0.080, SE = 0.02, p = 0.001), as shown in supplement 6. In siblings and controls, no significant association was found between smoking status and the DFAR task (Table 3).

**Table 3:** Results of linear mixed-effects model assessing the association between smoking status and DFAR task.

DFAR	Estimate	Patients SE	p-value	Estimate	Siblings SE	p-value	Estimate	Controls SE	p-value
Intercept	61.4	2.1	<0.0001	65.3	1.7	<0.0001	65.2	2.2	<0.0001
Smoking	1.96	0.6	0.003⁎	0.61	0.6	0.272	1.05	0.8	0.169

SFS=Social Functioning Scale.

⁎ Significant after Bonferroni correction (p < 0.007).

### 3.4 Cross-sectional associations between smoking status and theory of mind

We evaluated cross-sectional associations between smoking status and ToM, while correcting for covariates (Table 4). No significant associations between smoking status and both tasks were found in patients, siblings or healthy controls.

**Table 4:** Results of multiple regression models assessing the association between smoking status and respectively the Hinting task and the false belief score of the Picture Sequencing Task.

	B	Patients SE	p-value	B	Siblings SE	p-value	B	Controls SE	p-value⁎
Hinting									
Constant	16.6	0.5	<0.0001	17.9	0.3	<0.0001	18.0	0.3	<0.0001
Smoking	−0.17	0.2	0.375	−0.072	0.1	0.549	−0.020	0.1	0.886

PST - FB									
Constant	17.7	1.1	<0.0001	19.0	0.8	<0.0001	19.9	1.3	<0.0001
Smoking	0.52	0.4	0.248	0.098	0.3	0.756	−0.21	0.5	0.701

SFS=Social Functioning Scale.

⁎ Significant after Bonferroni correction (p < 0.007).

### 3.5 Multi-cross-sectional associations between smoking status and domains of social functioning

We evaluated multi-cross-sectional associations between smoking status and four domains of the Social Functioning Scale (Table 5). In patients, a significant negative association was found for the pro-social activities domain (estimate = −2.55, SE = 0.9, p = 0.004). Post-hoc analysis revealed a significant negative association between the number of cigarettes smoked per day and the pro-social activities domain in patients (estimate = −0.13, SE = 0.03, p < 0.0001), as shown in supplement 7. In siblings and controls, no significant association was found between smoking status and the domains of SFS.

**Table 5:** Results of linear mixed-effects models assessing the association between smoking status and domains of Social Functioning Scale.

SFS	Estimate	Patients SE	p-value	Estimate	Siblings SE	p-value	Estimate	Controls SE	p-value
Withdrawal									
Intercept	102.5	2.6	<0.0001	105.7	2.3	<0.0001	111.0	3.3	<0.0001
Smoking	−0.97	0.8	0.213	−0.75	0.7	0.291	−2.0	1.1	0.058

Interpersonal									
Intercept	122.8	4.0	<0.0001	128.5	2.6	<0.0001	132.1	2.9	<0.0001
Smoking	−0.43	1.2	0.725	0.10	0.8	0.896	−1.39	0.9	0.141

Recreation									
Intercept	116.1	3.4	<0.0001	120.3	2.9	<0.0001	119.5	3.7	<0.0001
Smoking	−2.68	1.0	0.010	−0.65	0.9	0.449	−1.87	1.2	0.120

Pro-social									
Intercept	116.3	3.0	<0.0001	116.3	2.4	<0.0001	123.1	3.1	<0.0001
Smoking	−2.55	0.9	0.004⁎	0.87	0.7	0.228	−0.75	1.0	0.459

SFS=Social Functioning Scale.

⁎ Significant after Bonferroni correction (p < 0.007).

### 3.6 Longitudinal associations between change in smoking behaviour and change in social cognition or functioning

Longitudinal associations were assessed for the DFAR task and pro-social activities domain of the SFS in patients. Between baseline and three-year follow-up, 49 patients (6.3%) quitted smoking and 27 patients (3.5%) started smoking. Between three-year and six-year follow-up, 44 patients (7.2%) quitted smoking and 23 patients (3.7%) started smoking (see supplement 8). No significant associations were found between change in smoking behaviour and change in emotion processing (as part of social cognition), nor between change in smoking behaviour and change in pro-social activities as part of social functioning (see supplement 9).

## 4 Discussion

### 4.1 Summary of findings

We explored the associations of tobacco smoking, social cognition and social functioning in a sample of patients with a non-affective psychotic disorder, unaffected siblings and healthy controls over a period of six years. Smoking prevalence was substantially higher in patients compared to siblings and controls. With respect to social cognition, we found that smoking patients outperformed their non-smoking peers on emotion processing to a small extent. In addition, a dose-response relationship was found for higher number of cigarettes smoked per day and better emotion processing. Regarding ToM (Hinting task and PST), no differences were found between smoking and non-smoking patients. Notably, we found a negative association between smoking status and the pro-social activities domain (as part of social functioning) in the patient group. Also, severity of smoking was negatively associated with pro-social activities. No significant associations between smoking status and social cognition nor social functioning were found in siblings or controls. Furthermore, longitudinal analyses showed no significant associations between change in smoking behaviour and change in social cognition nor functioning. Regarding associations between social cognition and social functioning, the PST was correlated to the interpersonal behaviour and to the recreational activities domain of the SFS. Smoking behaviour did not seem to have a moderating role.

### 4.2 Findings in previous research

Two previous cross-sectional studies (Reed et al., 2016; Sánchez-Gutiérrez et al., 2018) did not find an association between smoking and social cognition in patients with psychosis, which is partially in contrast with our results, as we found an association between smoking and emotion processing but not between smoking and ToM. However, both studies evaluated global social cognition instead of evaluating separate social cognition domains (the GEOPTE Scale of Social Cognition for Psychosis (Sanjuan et al., 2003) and the Mayer–Salovey–Caruso Emotional Intelligence Test (Mayer et al., 2003), respectively).

We found that pro-social activities, involving passive or active interactions with other people, were negatively associated with smoking in patients. This is in line with a longitudinal study which found that adolescent smoking predicted poor social functioning in the general population (Fluharty et al., 2018). However, we found no significant association between smoking and social functioning in siblings and healthy controls after applying Bonferroni correction. Studies evaluating this relationship in psychosis are scarce. Iasevoli et al. (2013) found that in patients with *therapy-resistant* schizophrenia, tobacco smoking was not associated with poorer social functioning. We found no effect of change in smoking behaviour on change in social cognition or functioning. The failure to find such an effect may be explained by lack of power as only approximately 10% of all participants changed their smoking behaviour. To the best of our knowledge, no previous studies in participants with a psychotic disorder evaluated the association between change in smoking behaviour and change in social cognition or functioning.

### 4.3 Interpretation and proposed mechanisms

Mechanisms explaining the association between smoking and slightly better emotion processing are unclear. A moderating effect of personality could be an explanation. Extraversion has been shown to be related to smoking (Hakulinen et al., 2015) and it has also been associated with better emotional perception in the healthy population. In patients with psychosis, one study (Kirihara et al., 2012) found that abnormal neurophysiological activity during emotion processing was associated with lower levels of extraversion. Thus, enhanced social cognition could be driven by the personality trait extraversion, which is also associated with smoking.

In understanding how smoking is associated with poor social functioning, patients' coping mechanisms may play a role. Patients with a psychotic disorder have been shown to use more ineffective coping skills, such as a palliative reaction (namely looking for unhealthy distraction) (van Dijk et al., 2019) or avoidance (remove oneself from difficult situations) (Phillips et al., 2009). This may explain the association between smoking and the avoidance of social situations.

Social support may also contribute to the association between smoking and participating in social activities. In the last decades, smoking has been more and more abandoned from daily social life. If smoking and its negative side-effects are associated with a decrease in social support, this may result in reduced possibilities to participate in social activities, and thus, worse social functioning. Lower pro-social activity scores might also be due to external factors, for example by the fact that people are avoiding smoking individuals. Further research is necessary to assess possible interaction effects.

Further research is necessary to assess possible interaction effects. Partially in contrast with abovementioned possible explanation, patients with a psychotic disorder seem to report greater social facilitation from smoking compared to normal controls (Kelly et al., 2012).

The fact that we found a slight positive association between smoking and emotion processing and a negative association between smoking and pro-social activities (as part of social functioning) may appear counterintuitive as social cognition and social functioning have been found to be associated (Fett et al., 2011). However, some authors (Addington et al., 2005) found that poor social functioning was independent of cognitive impairments in patients with a first-episode psychosis and in the current study no significant associations were found between emotion processing and social functioning.

Noteworthy, associations were assessed at different assessment periods. Whereas the association with social cognition was assessed at baseline and three years, social functioning was measured at three and six years of follow-up. Given the small percentage of participants who quit smoking, higher impairment in social functioning may reflect long-term negative outcome. Furthermore, nicotine may have acute positive effects on social cognition and more substantial long-term negative effects on social functioning. Unfortunately, in the current study cigarette breaks and nicotine intake were not registered. Hence, no conclusions can be drawn regarding possible acute effects.

Second, within the domain of social cognition, other tasks than emotion processing are possibly closer related to social functioning. In contrast to previous reported associations between social cognition and social functioning (Fett et al., 2011), we found no association with emotion processing and only small positive associations with ToM. With only a small negative correlation with the PST in siblings and no moderating effect of smoking, these findings do not add to the understanding of found opposite associations with the DFAR and SFS, respectively. Moreover, prospective change analyses revealed no associations between smoking and social outcomes.

Regarding the associations between smoking and respectively the DFAR task and the pro-social activities domain of the SFS, the results must be interpreted with some caution. Estimates are of small size, although they remain significant when controlling for numerous covariates.

### 4.4 Strengths and limitations

The strengths of this study include the large sample size, the presence of two comparison groups and prospective repeated measures, adding multi-cross-sectional and change results to existing literature. Furthermore, Bonferroni corrections were used minimizing the risk of type I errors.

The study has several limitations. First, no explorative overall analysis was done because of the comparative approach including siblings and controls as comparison groups. Second, a few linear mixed-effects models did not reach convergence. However, sensitivity analyses were done which revealed similar results as the primary model. Third, the degree of tobacco use was administered using the CIDI, which has a scope of 12 months. Hence, a proper cumulative (lifetime) measurement is not included in the CIDI. Although an association between pack years of tobacco smoking and cognitive deficits in the general population has been proposed (Sabia et al., 2012), we could not correct for the effect of lifetime smoking in the current study. Fourth, our sample was relatively young. No conclusions can be drawn regarding chronic or life-time effects of cigarette use. Findings in the current study could underestimate cumulative brain damage caused by smoking (i.e. induced oxidative stress, inflammation, etc.), which have been associated with cognitive decline. Nonetheless, participants were followed for a total period of 6 years and negative associations between smoking and social functioning were evaluated on this time point. Fifth, only two social cognitive domains (emotion processing and ToM) were evaluated while social cognition consists of four domains (van Hooren et al., 2008). Sixth, DFAR mean scores of siblings and controls are higher than those of patients, possibly creating a ceiling effect in the first groups. Seventh, the SFS is a self-rated instrument, which could be the cause of low ecological validity of the activity dimension (including the pro-social and recreational activities domains) (Schneider et al., 2017). Eighth, due to the observational design of the current study, reversed causality and residual confounding cannot be ruled out. Finally, the GROUP-cohort includes a relatively high-functioning sample of patients (Korver et al., 2012). This could restrict the generalizability of findings to other sample of patients with psychotic disorders.

### 4.5 Clinical implications

Although smoking patients performed slightly better on one task of social cognition, their participation in pro-social activities was reduced compared to their non-smoking peers. Poor social functioning is already of great concern in patients with a psychotic disorder (Addington et al., 2008), regardless of smoking. The knowledge that smoking is associated with reduced participation in social activities, combined with the well-known detrimental effects of smoking, emphasizes the importance to cease smoking as well as to provide the opportunity to participate in pro-social activities for patients with a non-affective psychotic disorder.

## CRediT authorship contribution statement

All authors are responsible for reported research and all authors have participated in the concept and design; analysis and interpretation of data; drafting or revising of the manuscript, and they have all approved the manuscript as submitted.

## Role of the funding source

The infrastructure for the GROUP study is funded through the Geestkracht Programme of the Dutch Health Research Council (Zon-Mw, grant number 10-000-1001), and matching funds from participating pharmaceutical companies (Lundbeck, AstraZeneca, Eli Lilly, Janssen Cilag) and universities and mental health care organizations (Amsterdam: Academic Psychiatric Centre of the Academic Medical Center and the mental health institutions: GGZ Ingeest, Arkin, Dijk en Duin, GGZ Rivierduinen, Erasmus Medical Centre, GGZ Noord Holland Noord. Groningen: University Medical Center Groningen and the mental health institutions: Lentis, GGZ Friesland, GGZ Drenthe, Dimence, Mediant, GGNet Warnsveld, Yulius Dordrecht and Parnassia psycho-medical center The Hague. Maastricht: Maastricht University Medical Centre and the mental health institutions: GGzE, GGZ Breburg, GGZ Oost-Brabant, Vincent van Gogh voor Geestelijke Gezondheid, Mondriaan, Virenze riagg, Zuyderland GGZ, MET ggz, Universitair Centrum Sint-Jozef Kortenberg, CAPRI University of Antwerp, PC Ziekeren Sint-Truiden, PZ Sancta Maria Sint-Truiden, GGZ Overpelt, OPZ Rekem. Utrecht: University Medical Center Utrecht and the mental health institutions Altrecht, GGZ Centraal and Delta).

## Declaration of competing interest

The authors have declared that there are no conflicts of interest in relation to the subject of this study.

## Appendix A Supplementary data

Supplementary material Image 1
