# Supplementary material for: The association between smoking behaviour, social cognition and social functioning in patients with a non-affective psychotic disorder: A prospective follow-up study
Source: Schizophr Res Cogn. 2021 Jun 30;26:100206. doi: 10.1016/j.scog.2021.100206 (PMC8259295; doi:10.1016/j.scog.2021.100206)
Supplement: Supplementary file 1 — Supplementary material [file mmc1.docx]

**SUPPLEMENTARY APPENDIX**

This appendix formed part of the original submission.

**Supplement to:**

*The association between smoking behaviour, social cognition and social functioning in patients with a non-affective psychotic disorder: A prospective follow-up study.*

Dekker TEG*, Van der Heijden HS*, Schirmbeck NF, Bartels-Velthuis AA, Van Amelsvoort T, Simons CJP, De Haan L, Vermeulen JM.

* Shared first author

GROUP Investigators not listed as main author: Richard Bruggeman, Wiepke Cahn and Jim van Os.

**Index:**

- Supplement 1: *page 2*
  Participants with follow-up assessment by site
- Supplement 2: *page 3*
  Participants with missing data on covariates at baseline
- Supplement 3: *page 4*
  Participants with missing data on outcome variables per period
- Supplement 4: *page 5*

Results of Spearman’s correlations between domains of the SFS and respectively the DFAR task and PST

- Supplement 5: *page 6*

Results of partial correlations assessing a possible moderating effect on the correlation between domains of the SFS and PST

- Supplement 6: *page 7*

Results of linear mixed-effects model assessing the association between the
number of cigarettes smoked per day and DFAR task

- Supplement 7: *page 8*

Results of linear mixed-effects model assessing the association between the
number of cigarettes smoked per day and pro-social activities in SFS

- Supplement 8: *page 9*
  Available data on change in smoking behaviour per subgroup over time
- Supplement 9: *page 10*
  Longitudinal results from multiple regression models regarding change in
  smoking status and change in social cognition and functioning in patients

**Supplement 1.
Participants with follow-up assessment by site**

|  | Baseline | | | 3 year | | | 6 year | | |
| --- | --- | --- | --- | --- | --- | --- | --- | --- | --- |
| *Site* | *Patients* | *Siblings* | *Controls* | *Patients* | *Siblings* | *Controls* | *Patients* | *Siblings* | *Controls* |
| Amsterdam | 275 | 253 | 100 | 210 | 200 | 80 | 172 | 171 | 56 |
| Groningen | 259 | 270 | 83 | 217 | 213 | 77 | 192 | 193 | 70 |
| Maastricht | 301 | 287 | 211 | 218 | 194 | 157 | 172 | 156 | 125 |
| Utrecht | 239 | 237 | 155 | 153 | 185 | 114 | 118 | 147 | 92 |
| Total | 1074 | 1047 | 549 | 798 | 792 | 428 | 654 | 667 | 343 |
| Of all participants, 46 patients, 10 siblings and 41 controls were excluded on baseline due to missing data. | | | | | | | | | |

**Supplement 2.
Participants with missing data on covariates at baseline**

| *Variable* | *Patients* | *Siblings* | *Controls* |
| --- | --- | --- | --- |
| Education in years | 62 (5.8%) | 35 (3.3%) | 14 (2.6%) |
| PANSS | 44 (4.1%) | n.a. | n.a. |
| CAPE | n.a. | 126 (12.0%) | 27 (4.9%) |
| PAS<12 | 143 (13.3%) | 77 (7.4%) | 17 (3.1%) |
| Cannabis use | 120 (11.2%) | 98 (9.4%) | 24 (4.4%) |
| N (%) = missing at baseline. PANSS=Positive and Negative Syndrome Scale. CAPE=Community Assessment of Psychic Experience, frequency subscales. PAS <12=Premorbid Adjustment Scale, up to 11 years old. | | | |

**Supplement 3.
Participants with missing data on outcome variables per period**

|  | Baseline | | | 3 year | | | 6 year | | |
| --- | --- | --- | --- | --- | --- | --- | --- | --- | --- |
| *Outcome variable* | *Patients* | *Siblings* | *Controls* | *Patients* | *Siblings* | *Controls* | *Patients* | *Siblings* | *Controls* |
| DFAR | 119 | 78 | 38 | 389 | 329 | 158 | n.a. | n.a. | n.a. |
| Hinting | 69 | 27 | 11 | n.a. | n.a. | n.a. | n.a. | n.a. | n.a. |
| PST – FB | n.a. | n.a. | n.a. | n.a. | n.a. | n.a. | 489 | 381 | 216 |
| SFS | n.a. | n.a. | n.a. | 358 | 278 | 137 | 503 | 380 | 206 |
| DFAR=Degraded Facial Affect Recognition. PST – FB=Picture Sequencing Task – false belief score. SFS=Social Functioning Scale. | | | | | | | | | |

**Supplement 4.**

**Results of Spearman’s correlations between domains of the SFS and respectively the DFAR task and PST**

| *DFAR* | Patients  ρ p-value | | Siblings  ρ p-value | | Controls  ρ p-value | |
| --- | --- | --- | --- | --- | --- | --- |
| Withdrawal | 0.054 | .158 | 0.048 | .211 | -0.003 | .947 |
| Interpersonal | 0.079 | .040 | 0.012 | .762 | 0.026 | .607 |
| Recreation | 0.019 | .616 | -0.028 | .467 | -0.089 | .079 |
| Pro-social | 0.047 | .224 | 0.060 | .113 | 0.073 | .148 |
| *PST - FB* |  |  |  |  |  |  |
| Withdrawal | -0.022 | .602 | -0.037 | .353 | 0.029 | .601 |
| Interpersonal | 0.124 | .003* | 0.035 | .372 | 0.147 | .007* |
| Recreation | 0.090 | .028 | -0.117 | .003* | -0.034 | .543 |
| Pro-social | 0.078 | .057 | -0.085 | .032 | 0.135 | .014 |
| SFS=Social Functioning Scale. DFAR=Degraded Facial Affect Recognition. PST-FB=Picture Sequencing Task - false belief score. *significant at a p-value of .01 | | | | | | |

**Supplement 5.**

**Results of partial correlations assessing a possible moderating effect on the correlation between domains of the SFS and PST**

| *PST - FB* | Patients  ρ p-value | | Siblings  ρ p-value | | Controls  ρ p-value | |
| --- | --- | --- | --- | --- | --- | --- |
| Interpersonal | 0.123 | .003* | n.a. | n.a. | 0.144 | .009* |
| Recreation | n.a. | n.a. | -0.117 | .003* | n.a. | n.a. |
| SFS=Social Functioning Scale. PST-FB=Picture Sequencing Task - false belief score. *significant at a p-value of .01 | | | | | | |

**Supplement 6.
Results of linear mixed-effects model assessing the association between the number of cigarettes smoked per day and DFAR task**

|  | Patients | | |
| --- | --- | --- | --- |
| *DFAR* | Estimate | SE | p-value |
| Intercept | 62.1 | 2.0 | <0.0001 |
| Number of cigarettes/day | 0.080 | 0.02 | 0.001 |
| DFAR=Degraded Facial Affect Recognition. *significant at a p-value of .007 | | | |

**Supplement 7.
Results of linear mixed-effects model assessing the association between the number of cigarettes smoked per day and the pro-social activities domain of the SFS**

|  | Patients | | |
| --- | --- | --- | --- |
| *Pro-social activities of SFS* | Estimate | SE | p-value |
| Intercept | 116.5 | 2.9 | <0.0001 |
| Number of cigarettes/day | -0.13 | 0.03 | <0.0001 |
| SFS=Social Functioning Scale. | | | |

**Supplement 8.**

**Available data on change in smoking behaviour**

| *Subgroup* | DFAR: Baseline – 3 year | SFS: 3 year – 6 year |
| --- | --- | --- |
| No smoker | 242 | 228 |
| Continue smoker | 460 | 320 |
| Quit smoker | 49 | 44 |
| Start smoker | 27 | 23 |
| Total | 778 | 615 |
| N = available data for calculating a change in smoking status; change scores for DFAR and SFS between baseline and three years of follow-up and between three and six years of follow-up were calculated. | | |

**Supplement 9.**

**Longitudinal results from multiple regression models regarding change in smoking status and change in social cognition and functioning**

|  | *Patients* | | | |
| --- | --- | --- | --- | --- |
| Effects | B | SE | p-value | N |
| *Degraded Facial Affect Recognition task (Baseline – 3 year)* | | | | |
| Constant | -2.300 | 4.097 | 0.575 | 509 |
| Ceased smoking | 1.901 | 1.972 | 0.336 |  |
| Constant | 6.946 | 4.303 | 0.108 | 269 |
| Started smoking | -0.250 | 0.877 | 0.776 |  |
| *Social Functioning Scale – pro-social activities (3 year – 6 year)* | | | | |
| Constant | -0.627 | 4.696 | 0.894 | 364 |
| Ceased smoking | 0.245 | 2.316 | 0.916 |  |
| Constant | -0.186 | 3.927 | 0.962 | 251 |
| Started smoking | 0.429 | 0.806 | 0.595 |  |
